# Post-traumatic Takotsubo Cardiomyopathy in a Pediatric Patient: A Rare Case and Diagnostic Challenge

**DOI:** 10.7759/cureus.74802

**Published:** 2024-11-29

**Authors:** Adil Zyani, Oualid Mzaalak Tazi, Rajae Alkouh, Ounci Es-Saad, Smael Labib

**Affiliations:** 1 Anesthesia and Critical Care, Mohammed VI University Hospital, Tangier, MAR; 2 Faculty of Medicine and Pharmacy of Tangier, Abdelmalek Essaâdi University, Tangier, MAR

**Keywords:** echocardiogram, general trauma surgery, stress-induced cardiomyopathy, takotsubo cardiomyopathy, trauma-induced cardiomyopathy

## Abstract

Takotsubo cardiomyopathy (TTC), also known as stress-induced cardiomyopathy, is a rare condition in children that causes acute, severe, but often reversible systolic dysfunction of the left ventricle. Physical trauma is a recognized trigger, although distinguishing TTC from myocardial contusion in pediatric trauma cases can be challenging due to overlapping clinical features. We present the case of a six-year-old boy involved in a high-impact motor vehicle collision. The patient initially presented with multiple traumatic injuries, including fractures of the skull, ribs, and right upper extremity, as well as pulmonary contusions. After initial stabilization in the pediatric intensive care unit (PICU), he developed hemodynamic instability six hours postoperatively, with elevated troponin levels suggesting myocardial contusion. Echocardiography later revealed severe left ventricular dysfunction with apical akinesia and basal hyperkinesis, hallmark findings of TTC. Inotropic support was switched from dobutamine to milrinone to avoid exacerbating catecholamine-induced myocardial stress. Over seven days, the patient’s left ventricular function normalized, with an ejection fraction of 55%, and he was discharged on day 15 in stable condition. This case highlights the importance of early recognition of TTC in pediatric trauma patients, where echocardiography and cautious use of inotropic agents can ensure optimal outcomes.

## Introduction

Takotsubo cardiomyopathy (TTC), or stress-induced cardiomyopathy, was first described in 1990 in Japan. The condition derives its name from the resemblance of the left ventricular shape during systolic dysfunction to a “Takotsubo,” a Japanese octopus trap. TTC is characterized by transient and reversible left ventricular systolic dysfunction that mimics acute coronary syndrome (ACS) but occurs without coronary artery obstruction [1]. It is typically triggered by emotional or physical stress, which causes a catecholamine surge, resulting in myocardial stunning and the characteristic apical ballooning [2].

In adults, TTC predominantly affects postmenopausal women and accounts for 1-2% of suspected ACS cases [3]. Its occurrence in pediatric populations, however, is rare and underreported [4,5]. While emotional stress is a common trigger in adults, physical trauma, such as severe accidents or brain injuries, has been increasingly recognized as a cause of pediatric TTC. Due to overlapping features with conditions such as myocarditis or myocardial contusion, pediatric TTC is often misdiagnosed [4].

In the context of trauma, distinguishing TTC from myocardial contusion is critical. Both conditions share overlapping features, including elevated cardiac biomarkers and regional wall motion abnormalities, yet they require different management strategies. Echocardiography plays a pivotal role in diagnosing TTC, revealing the hallmark apical ballooning pattern that is absent in myocardial contusion [5]. This case report highlights a rare instance of trauma-induced TTC in a pediatric patient following a high-impact motor vehicle accident, initially misdiagnosed as a myocardial contusion. It emphasizes the importance of careful diagnostic evaluation and the critical role of echocardiography in guiding appropriate management.

## Case presentation

A six-year-old male, weighing 24 kg, presented to the emergency department following a high-impact collision between a cargo tricycle and a van, which tragically resulted in the death of two individuals at the scene. Upon admission, the patient was afebrile and pale, with normal capillary blood glucose levels. He was neurologically stable, scoring 15 on the Glasgow Coma Scale (GCS), and was hemodynamically stable. Respiratory examination revealed tachypnea at 37 breaths per minute, with no significant pleuropulmonary abnormalities noted. The patient had no notable past medical history, and there had never been any clinical indication for cardiovascular evaluation.

**Figure 1:**
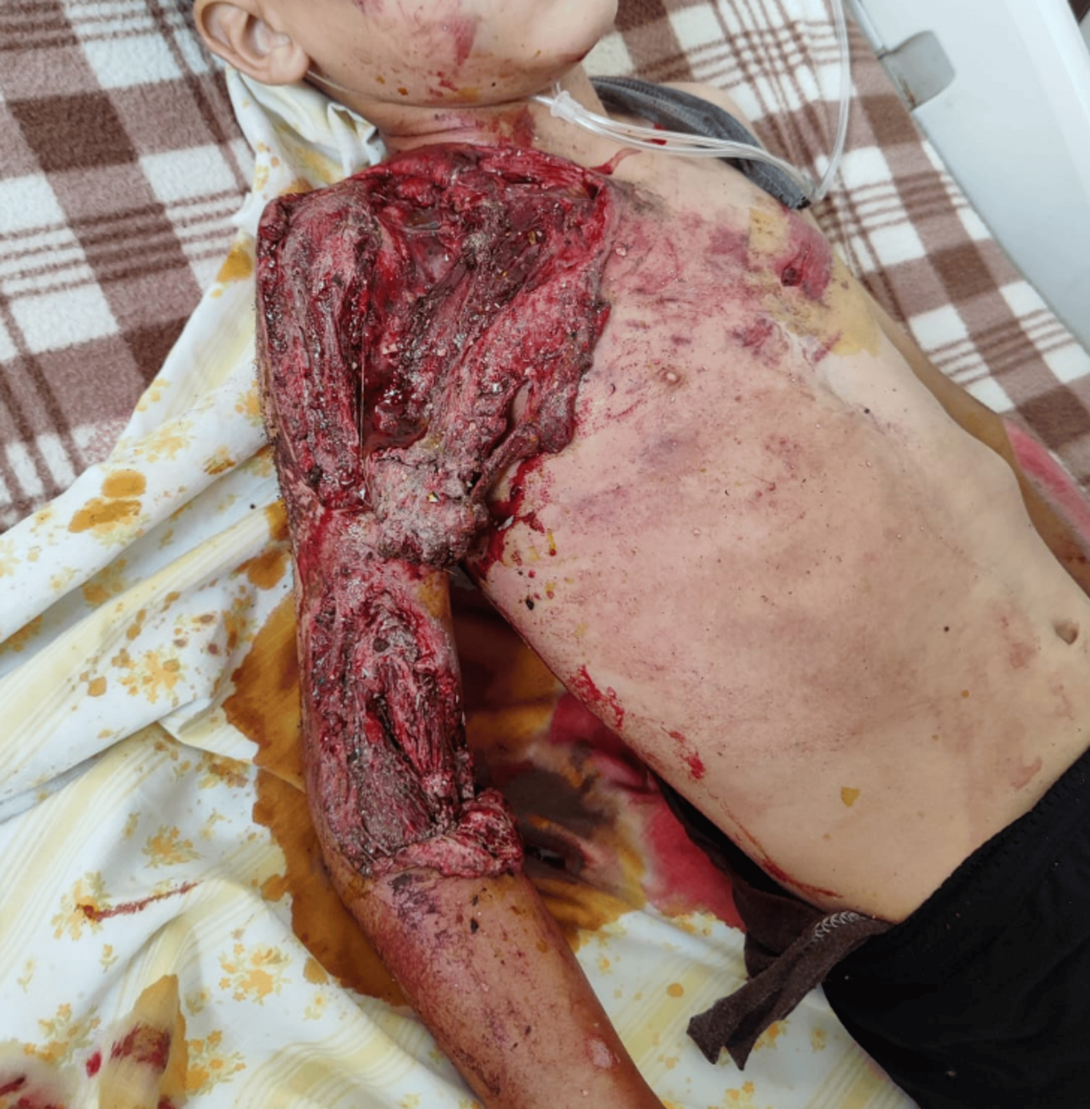
Image showing large soft tissue loss in the right upper limb.

On physical examination, the patient exhibited significant soft tissue loss in the right upper limb, along with multiple abrasions on the trunk, face, and limbs (Figure 1). Musculoskeletal examination revealed an open fracture of the right clavicle and deformities in the right elbow and forearm.

**Figure 2:**
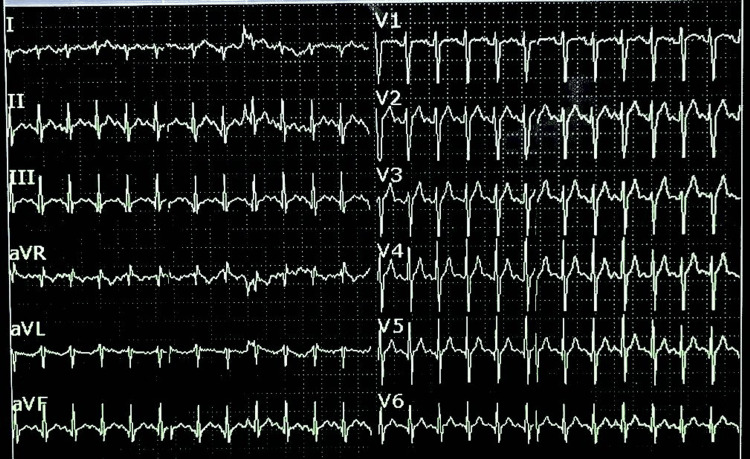
EKG showing sinusal tachycardia with no evidence of ST-segment changes or repolarization abnormalities.

The initial radiological assessment revealed pneumocephalus with simple fractures of the right parietal and temporal bones, right-sided pulmonary contusions with fractures of the third and fourth ribs, and multiple fractures involving the facial bones, scapula, right clavicle, and both bones of the right forearm. The patient's electrocardiogram (EKG) showed sinus tachycardia without depolarizing abnormalities (Figure 2).

The patient was taken to the operating room for debridement and suturing of the soft tissue injuries under general anesthesia. During surgery, his hemodynamic status deteriorated, requiring norepinephrine at doses of up to 4 µg/kg/min. Postoperatively, he was transferred to the pediatric intensive care unit (PICU), where his condition initially stabilized. However, six hours later, the patient developed severe hypotension and oliguria, prompting a focused reassessment.

**Figure 3:**
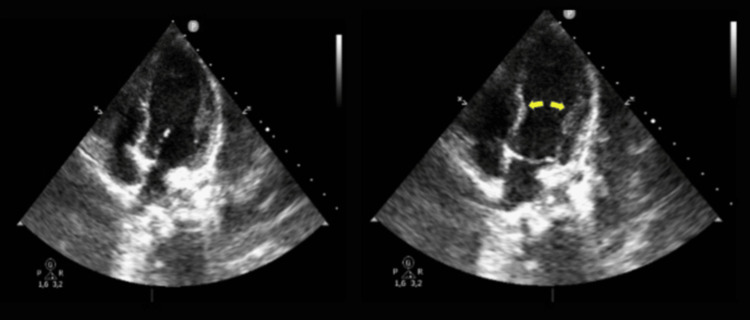
Image showing the characteristic apical ballooning of TTC.

Given the blunt chest trauma, elevated troponin levels (3,438 ng/L), and worsening hemodynamic instability, myocardial contusion was initially suspected. A focused assessment with sonography for trauma (FAST) revealed no significant pericardial or pleural effusions. An echocardiogram was subsequently performed, which revealed severe left ventricular dysfunction with apical akinesia and basal hyperkinesis, hallmark features of TTC, with an ejection fraction (EF) of 25% (Figure 3). These findings, along with the disproportionate elevation of troponin levels relative to the degree of myocardial injury expected in contusion, shifted the diagnosis to TTC.

Dobutamine was initially administered for inotropic support but was later replaced with milrinone due to concerns about the adverse effects of exogenous catecholamines in TTC. Over the subsequent days, the patient’s condition gradually stabilized, and his left ventricular function completely normalized with an EF of 55% by day seven of hospitalization. Troponin levels also decreased and returned to normal by day eight. The patient was discharged in stable condition on day 15 of hospitalization.

## Discussion

TTC, or stress-induced cardiomyopathy, is a transient condition characterized by sudden and reversible left ventricular dysfunction, predominantly affecting the apical and mid-segments, resulting in the distinctive "apical ballooning" appearance on echocardiography [1]. Although most commonly diagnosed in postmenopausal women, TTC is rare in pediatric patients and remains underrepresented in the literature compared to adults. Among pediatric cases, TTC predominantly affects adolescents, with physical trauma or neurological injury acting as primary triggers [4,5]. This highlights the importance of increased awareness of TTC in younger age groups, particularly following significant physical stressors.

The pathophysiology of TTC in both adults and children is believed to involve a catecholamine surge triggered by emotional or physical stress, leading to myocardial injury, microvascular dysfunction, and coronary spasm [5]. This catecholamine surge causes myocardial stunning, and while this mechanism is shared across age groups, children are more likely to be misdiagnosed with myocarditis or myocardial contusion due to the rarity of atherosclerosis in this population [6].

In our case, a six-year-old boy developed TTC following a high-impact motor vehicle collision, presenting diagnostic challenges due to overlapping features with myocardial contusion. Both conditions share common clinical and biological markers, such as chest pain, elevated cardiac biomarkers, and regional wall motion abnormalities [7]. However, key distinctions helped guide the diagnosis. While elevated troponin levels are commonly seen in both TTC and myocardial contusion, our case demonstrated a modest elevation of troponin disproportionate to the severity of left ventricular dysfunction seen on echocardiography. Biomarkers such as creatine kinase-MB (CK-MB) and troponins, while supportive, are non-specific and fail to reliably discriminate between ischemic and non-ischemic cardiac injuries, particularly in pediatric trauma patients [1]. Their role should, therefore, complement imaging findings rather than serve as a standalone diagnostic tool.

Echocardiography in our patient revealed apical ballooning with basal hyperkinesis, hallmark findings of TTC, which differ from the echocardiographic patterns typically associated with myocardial contusion. Myocardial contusion, caused by direct mechanical trauma, more commonly affects the right ventricle and atrium due to their anatomical position. This distinction was crucial in guiding the diagnosis in our case, emphasizing the indispensable role of echocardiography in trauma-related cardiac injuries.

Management of TTC in this case required careful consideration of inotropic support. While norepinephrine was initially used to stabilize hemodynamics, dobutamine was subsequently introduced for inotropic support. However, due to concerns about exacerbating catecholamine-induced myocardial dysfunction, dobutamine was replaced with milrinone [8,9]. Non-catecholaminergic inotropes, such as levosimendan and milrinone, are preferred in managing TTC due to their mechanisms that do not involve adrenergic stimulation. These agents align with current recommendations for TTC management and were instrumental in stabilizing our patient’s condition, allowing for recovery of left ventricular function [10].

The prognosis for TTC is generally favorable, particularly in pediatric patients, provided that the condition is promptly recognized and appropriately managed [3]. In contrast, severe myocardial injury from blunt trauma, such as myocardial contusion, carries a significantly higher mortality rate, especially when concomitant head injury is present. Such fatalities often occur at the site of the accident or during transportation [11]. In our case, the patient’s left ventricular function completely normalized with an ejection fraction of 55% by the seventh day of hospitalization, and he was discharged in stable condition after 15 days. This rapid recovery aligns with the expected course of TTC, which typically resolves over days to weeks with supportive care, as reported in pediatric cases where recovery has been documented within three days to three months of presentation [3,12]. Despite the acute severity of TTC, early recognition, careful monitoring, and appropriate intervention ensure a good prognosis in most cases.

In summary, our case illustrates the diagnostic challenges in distinguishing TTC from myocardial contusion in pediatric trauma patients. Echocardiography remains the most reliable diagnostic tool, while biomarkers should be interpreted with caution. Effective management involves avoiding catecholamines and favoring non-catecholaminergic agents such as milrinone or levosimendan. Early recognition and appropriate management are key to achieving favorable outcomes in this rare condition.

## Conclusions

TTC is a rare but critical differential diagnosis in pediatric trauma cases presenting with cardiac dysfunction. While it shares clinical features with myocardial contusion, careful evaluation through echocardiography is crucial for distinguishing the two conditions. This case underscores the critical role of echocardiography in identifying TTC, as it can be easily misdiagnosed as myocardial contusion in trauma settings. Biomarkers, though non-specific, provide supportive evidence when combined with imaging findings. In this case, the rapid normalization of left ventricular function by day seven, and the resolution of troponin levels by day eight strongly supported the diagnosis of TTC. Early recognition and management tailored to the pathophysiology of TTC, particularly avoiding catecholamines and opting for agents such as milrinone or levosimendan, are essential for achieving optimal outcomes.

While the diagnosis was based on clinical and echocardiographic findings, the absence of advanced imaging modalities, such as cardiac MRI, represents a limitation and could have further corroborated the differentiation from myocardial contusion. Nevertheless, as demonstrated in this case, with appropriate supportive care, the prognosis for pediatric TTC is excellent, with full recovery of cardiac function typically observed within days to weeks.
